# 102. Efficacy and Effectiveness of High-Dose Influenza Vaccine in Older Adults by Age and Seasonal Characteristics: An Updated Systematic Review and Meta-Analysis

**DOI:** 10.1093/ofid/ofac492.180

**Published:** 2022-12-15

**Authors:** Jason K Lee, Gary K Lam, Rolan Vaisman, Kevin J Yin, Bruce T Seet, Matthew M Loiacono, Sandrine I Samson

**Affiliations:** Sanofi, Toronto, Ontario, Canada; Sanofi, Toronto, Ontario, Canada; Sanofi, Toronto, Ontario, Canada; Sanofi, Toronto, Ontario, Canada; Sanofi, Toronto, Ontario, Canada; Sanofi, Toronto, Ontario, Canada; Sanofi, Toronto, Ontario, Canada

## Abstract

**Background:**

High-dose influenza vaccine (HD-IIV) has been used extensively in older adults since its approval in 2009. Studies comparing HD-IIV to standard-dose influenza vaccines (SD-IIV) have demonstrated protection beyond influenza, including reduction of outcomes like cardiorespiratory and all-cause hospitalizations. This study is an update of previously-conducted reviews of the relative vaccine efficacy/effectiveness (rVE) of HD-IIV vs. SD-IIV in adults ≥65 years against influenza-associated outcomes from 2009-2020, with sub-analyses by seasonal and recipient characteristics.

**Methods:**

An updated systematic review and meta-analysis was conducted for studies assessing the rVE of HD-IIV against probable/laboratory-confirmed influenza-like illness (ILI), emergency department (ED) and/or hospital admissions in adults ≥65 years. Results from individual seasons – stratified by outcome, subject characteristics, and influenza season, were meta-analyzed to estimate pooled rVEs of HD-IIV.

**Results:**

19 studies from 11 consecutive seasons with over 45 million HD-IIV or SD-IIV recipients were meta-analyzed. Overall, HD-IIV demonstrated improved protection vs. SD-IIV against ILI (rVE=14.3%, 95%CI:4.2-23.3%), HD-IIV was more effective at preventing hospital/ED visits due to influenza (rVE=10.4%, 95%CI:6.8-13.9%), and hospitalizations due to influenza (rVE=11.2%, 95%CI:7.4-14.8%), pneumonia (rVE=27.3%, 95%CI:15.3-37.6%); pneumonia and influenza (rVE=13.4%, 95%CI:7.3-19.2%); respiratory (rVE=14.3%, 95%CI:8.5-20.0%), cardiovascular (rVE=13.1%, 95%CI:10.5-15.7%), and cardiorespiratory events (rVE=17.9%, 95%CI:15.0-20.8%); and all-causes (rVE=8.4%, 95%CI:5.7-11.0%). Pooled rVEs were similar in sub-analyses by dominant strain, antigenic match, and study type/setting. While all adults 65+ benefited from the use of HD-IIV, age-stratified analyses of the rVE of HD-IIV suggested additional relative benefit with increasing age.

**Conclusion:**

Evidence over 11 consecutive influenza seasons from both randomized and observational studies suggests HD-IIV was consistently more effective than SD-IIV at reducing influenza and associated serious outcomes irrespective of recipient age and characteristics of the influenza season.

**Disclosures:**

**Jason K. Lee, MS, MBiotech**, Sanofi: Employee|Sanofi: Stocks/Bonds **Gary K. Lam, PharmD, RPh**, Sanofi: Employee **Rolan Vaisman, PharmD, RPh**, Sanofi: Employee **Kevin J. Yin, MD, MPH, PhD**, Sanofi: Employee|Sanofi: Stocks/Bonds **Bruce T. Seet, PhD, MBA**, Sanofi: Employee|Sanofi: Stocks/Bonds **Matthew M. Loiacono, PhD, MSc**, Sanofi: Employee|Sanofi: Stocks/Bonds **Sandrine I. Samson, PhD**, Sanofi: Employee|Sanofi: Stocks/Bonds.

